# The interaction protein of SORBS2 in myocardial tissue to find out the pathogenic mechanism of LVNC disease

**DOI:** 10.18632/aging.203841

**Published:** 2022-01-20

**Authors:** Chunyan Li, Yang Zheng, Ying Liu, Guo Hong Jin, Haizhou Pan, Fenghui Yin, Jun Wu

**Affiliations:** 1 Department of Clinical Laboratory, Beijing Jishuitan Hospital, Beijing 100035, China; 2 Department of Laboratory Medicine, Peking University Fourth School of Clinical Medicine, Beijing Jishuitan Hospital, Xicheng, Beijing 100035, China; 3 Department of Orthopaedics, Fourth Medical Center of PLA General Hospital, People's Republic of China, National Clinical Research Center for Orthopedics, Sports Medicine and Rehabilitation, Beijing 10048, China; 4 Department of Cardiovascular Surgery, The First Affiliated Hospital of Zhejiang University, Hangzhou 310014, China; 5 The Third People’s Hospital of Chengdu, Chengdu 610031, China

**Keywords:** SORBS2, LVNC, YWHAQ

## Abstract

Background: Left ventricular noncompaction cardiomyopathy (LVNC) is a cardiac disorder characterized by an excessive trabecular meshwork of deep intertrabecular recesses within the ventricular myocardium. Sorbin and SH3 domain-containing protein 2 (SORBS2) converges on the actin and microtubule cytoskeleton. Here, we investigated the proteins interacting with SORBS2 to elucidate the pathogenic mechanism of LVNC. As reported in previous studies, SORBS2 enhances the occurrence of LVNC by potentiating heart failure, but the specific mechanism remains unclear.

Methods: Building from our previous finding of elevated SORBS2 levels in LVNC hearts, we screened for proteins interacting with SORBS2 by proteomics and conducting IP experiments. Co-IP and immunofluorescence were used to verify the effects.

Results: We selected several proteins with high scores and high coverage that could be closely related to SORBS2 according to earlier reports showing a correlation with LVNC for verification. We finally obtained several proteins that were related to the pathogenesis of LVNC and also interacted with SORBS2, such as α-actinin, β-tubulin, MYH7, FLNA, MYBPC3, YWHAQ and DES, and YWHAQ was the most associated.

Conclusions: We focused on the YWHAQ protein, and we identified a novel mechanism through which SORBS2 interacts with YWHAQ, having a negative effect on the cell cycle, potentially leading to LVNC.

## INTRODUCTION

Left ventricular noncompaction cardiomyopathy (LVNC) is a clinically heterogeneous disorder, currently classified as a primary genetic cardiomyopathy by the American Heart Association [1]. It is a cardiac disorder characterized by an excessive trabecular meshwork of deep intertrabecular recesses in the ventricular myocardium. Studies have shown that the incidence of LVNC is highly variable, where the main reason is that insufficient understanding of LVNC may cause missed diagnosis and misdiagnosis in clinical practice. Therefore, clinicians face great challenges in the diagnosis and treatment of LVNC. The most common clinical presentations of LVNC are congestive heart failure, cardiac arrhythmia and thromboembolism [2]. Nowadays, diagnostic criteria have been generated from different imaging modalities [3–7] on the basis of the morphological appearance of the myocardium. Novel pathogenic LVNC genes must be identified because of the unclear mechanism and small number of causes of LVNC.

Sorbin and SH3 domain-containing protein 2 (SORBS2) is an adaptor protein and a member of the SOHO family. SORBS2 shows high expression in the myocardium and is localized in the Z-bands of mature myofibrils [8], but its cardiac-specific function is unknown. Emerging evidence demonstrates that SORBS2 contributes to microtubule densification, also altering the distribution of JP2, leading to excitation-contraction (E-C) coupling, and may cause heart failure [9]. Studies have demonstrated that defective E-C coupling is a hallmark of heart failure [10–12]. Whether the high expression of SORBS2 is the main pathogenic mechanism of LVNC remains to be investigated.

The pathogenic causes and pathogenesis of LVNC involve many factors such as embryo development, genetic variation and so on [3, 4]. In the process of cardiac development, because of genetic abnormalities and cell cycle arrest, cell development is blocked and the densification process is impaired, resulting in the persistence of protruding trabecular muscle and trabecular space, causing the development of spongiform endocardial ventricular muscle.

14-3-3 proteins are highly conserved, ubiquitous in all eukaryotic cells, and regulated by a family of proteins encoded by a single gene family in most species. They are involved in almost all physiological processes in life, and various 14-3-3 proteins have been found in various tissues and cells. Previous studies have indicated that as the first signaling molecule bound to serine/threonine phosphate, the 14-3-3 protein plays an important role in cellular signal transduction, especially in the direct regulation of protein kinase and protein phosphorylase activity, and it is called the "bridge protein" of protein-protein interaction. It can combine with transcription factors to form complexes that regulate the expression of related genes. 14-3-3 proteins are involved in the cell cycle, apoptosis and cell signaling regulation [13].

Our studies in hESC-derived cardiomyocytes (hESC-CMs) provided a new finding that SORBS2 interacts with YWHAQ regardless of the position or function. Specifically, data statistics have shown that some proteins have a very high level of relevance with SORBS2 in normal tissues. *In vitro*, the hESC-CMs with overexpressed SORBS2, similarly to normal heart tissues, showed that SORBS2 and YWHAQ interacted at the same position. Moreover, increased expression of SORBS2 caused a decrease in YWHAQ expression. These data collectively indicated that the increased levels of SORBS2 contribute to decreased YWHAQ, which may be involved in the pathogenesis of LVNC by interfering with the cell cycle.

## MATERIALS AND METHODS

### Mass spectrometry identification experiment

After silver staining and cutting adhesive, each adhesive strip was enzymatically decomposed. The IP products were detected by mass spectrometry on the computer, and then combined to search the database. The strip was destained and dried at room temperature (RT). Reduction/alkylation was carried out, and NH_4_HCO_3_ was added, followed by shaking for 5 min. The solution was discarded and strips dried at RT. Trypsin solution was added and incubation was at 4° C for 60 min. Overnight enzymatic hydrolysis was at 37° C. Extract enzyme-hydrolyzed polypeptide. Centrifugal concentration drying. Sample loading processing. After vacuum drying, 0.1% FA was added to redissolve the sample, and 1-2 μg of sample were put on the instrument. The separation was performed with EASY nLC 1200 (Thermo Scientific, USA) using a self-filled Trap column (C18, 5 μm, 100 μm×2 cm) and an analysis column (C18, 1.9 um, 75 μm×20 cm) at a flow rate of 200 NL/min. The mass spectrometer was Orbitrap Fusion Lumos (Thermo Scientific, USA). The Data Dependent Acquisition (DDA) mode was adopted for the detection of tandem mass spectrometry. Full sweep resolution was 60,000 (FWHM), and the mass/charge ratio range was set at M/Z 375-1600, and collision energy was set at 30% in HCD fragmentation mode.

### Screening for proteins that interact with SORBS2

The myocardial tissue of normal adult mice at 12 weeks of age was analyzed by mass spectrometry. GO (Biological Process, Molecular Function, Cellular Component) analysis and Pathway analysis were performed for the specifically detected proteins, and bioinformation annotation and analysis were performed at two levels. In this way, more important proteins were selected for further study. The interacting proteins were screened by mass spectrometry.

### Western blotting

The cells were harvested and washed twice with PBS. They were then lysed with cold RIPA and centrifuged at 12,000 g at 4° C for 10 min. The supernatants were collected and assayed for protein by the BCA method. The tissue and cell homogenates were separated by SDS-PAGE (4-12% gradient) and transferred onto a PVDF membrane. The PVDF membranes were blocked by TBS-T with 5% milk for about 2 h. The PVDF membranes were then incubated with antibodies overnight at 4° C and incubated with secondary HRP-IgG antibodies (CST Biotechnolog, USA) for 2 h at RT.

The primary antibodies were as follows: SORBS2 (1:1000; Proteintech); YWHAQ (1:1000; Proteintech); GAPDH (1:1000; Proteintech); β-tubulin (1:1000; CST).

### Immunofluorescence

Cells were cultured on glass coverslips with gelatin, fixed for 15 min in cold 4% paraformaldehyde, washed with PBS for 10 min, incubated with 5% normal goat serum (NGS; ZSGB-BIO, Beijing, China) in PBS for 1 h at RT, and then washed with PBS for 10 min. Cells were then incubated with the primary antibody overnight at 4° C, washed with PBS for 10 min, incubated with IgG peroxidase-conjugated secondary antibody for 1 h, and washed with PBS for 10 min. The cells were examined with a confocal scanning laser microscope (Sp8; Leica, Germany). The immunohistochemistry procedure was similar to that described above. The myocardial samples were fixed in 10% neutral buffered formalin. They were paraffin embedded after using alcohol and xylene gradients for dehydration. They were then blocked with 0.05% H2O2.

Immunostaining was done with the following antibodies: SORBS2 (1:100; Proteintech) and sarcomeric α-actinin (1:100, Abcam), CLTC polyclonal antibody (1:100; Proteintech); β-tubulin (1:100;CST); YWHAQ (1:200;CST); Oct4A (1:100;CST); MLC-2V (1:100; Proteintech); CTNI (1:100; Proteintech); CTNT (1:100; Proteintech); Sox2 (1:100; CST); SSEA (1:100; CST); Alexa 488-conjugated goat anti-mouse (Yeasen, Shanghai, China); Alexa 488-conjugated goat anti-rabbit (Yeasen, Shanghai); Alexa 594-conjugated goat anti-mouse (Yeasen, Shanghai); and Alexa 594-conjugated goat anti-rabbit (Yeasen, Shanghai). The cell nuclei were counterstained with 0.1% 4’,6-diamidino-2-phenylindole (DAPI; Yeasen, Shanghai).

### Cell culture

The human embryonic stem cells were cultured in TeSR™-E8™ Basal Medium supplemented with TeSR™-E8™ media (StemCell Technologies, Cat. # 05990). Once the stem cells reached 70% confluency, they were then prepared to be induced to differentiate into hESC-CMs. RPMI1640 containing 3 μM CHIR-99021HCl (Selleck Chem, Cat. # S2924), and 2% B-27 minus insulin (Gibco, USA) as the culture medium was used to induce hESC. After 3 days, the cells were cultured in RPMI1640 containing 5 μM IWR-1-endo (Selleck Chem, S7086) and 2% B-27 minus insulin (Gibco). Two days later, the medium was replaced with RPMI1640 containing 2% B-27 minus insulin (Gibco). From then on, the medium was changed every 3 days with RPMI1640 plus 1× B-27 supplement. On day 16, collagenase type I (Sigma) was used to purify the cells, and the cells were then cultured in Dulbecco’s Modified Eagle’s Medium supplemented with 15% fetal bovine serum (Gibco) at 37° C and 5% CO_2_ in a humidified incubator, with the medium changed every day.

### Cell transfection

To demonstrate the capacity of SORBS2 in cardiomyocytes, we created two appropriate carriers HBLV-GFP-PURO-NC and HBLV-h-3*flag-GFP-PURO to disrupt the cardiomyocytes. We purchased the lentivirus from HANBIO and strictly followed the instructions. The lentivirus could transfect after the hESC reached nearly 70% confluence with 1×10^6^ viruses per well of a 6-well plate. At 48 h after transfection, green fluorescence intensity could be observed under a fluorescence microscope. The transfected target cells were then isolated to proceed with the next experiments.

### Co-immunoprecipitation

Co-IP assays were carried out using the 88804 Pierce Classic Magnetic IP/Co-IP Kit, Thermo [14]. The antibodies used were as follows: IP: rabbit polyclonal anti-SORBS2 (2 μg; Proteintech); rabbit polyclonal anti-YWHAQ (2 μg; Proteintech); IB: rabbit polyclonal anti-SORBS2 (1:1000; Proteintech); and rabbit monoclonal anti-YWHAQ (1:1000; CST). Heart tissues were lysed in ice-cold lysis buffer. Protein was collected by centrifugation at 12,000 rpm for 10 min at 4° C. The lysates were then incubated with 5 μg of antibodies pre-bound to protein A/G-Sepharose beads overnight at 4%. Beads were washed four times with ice-cold washing buffer, and eluted in SDS-PAGE sample repeat (50 mM Tris–HCl (pH 6.8), 2% SDS, 0.1% bromophenol blue, 10% glycerol, 10 mM dithiothreitol). The precipitates were subjected to immunoblotting with antibodies.

### Statistical analysis

All data were analyzed with SPSS Statistics 22 software. One-way analysis of variance (ANOVA) was used to evaluate the differences between the two groups. In all the analyses, P-value<0.05 and P-value<0.01 were considered indicative of a statistically significant difference.

## RESULTS

### Screening for proteins interacting with SORBS2 by mass spectrometry

**Figure 1:**
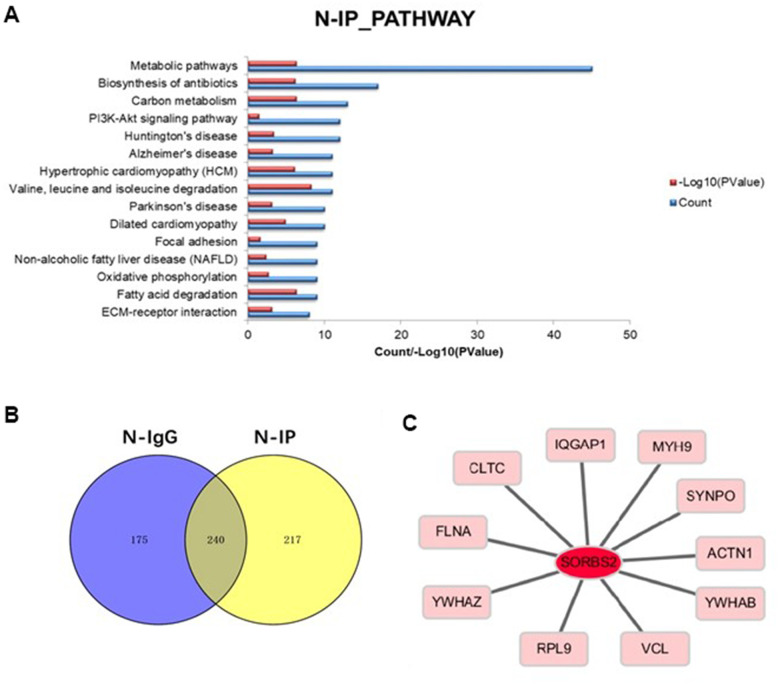
**Proteins interacting with SORBS2 were screened by IP experiment.** (**A**) IP experiment screened the signal pathways that were different from SORBS2. (**B**) The Venn diagram shows the difference between the two groups of IgG and IP. (**C**) Proteins interacting with SORBS2 were screened by bioinformatics database. The main enrichment pathway of the proteins interacting with SORBS2 was screened by IP experiments.

In this study, we performed mass spectrometric identification of SORBS2-interacting proteins in normal mouse cardiac muscle tissue and hESC-CMs. For the specifically detected proteins, GO (Biological Process, Molecular Function, Cellular Component) analysis and pathway analysis were performed, and biological information annotation was conducted at two levels (Figure 1A, 1B). Thus, more important proteins were singled out for further study. Therefore, we used the myocardial tissue to identify the interacting proteins by mass spectrometry (Figure 1C).

These data suggested that as a specific protein up-regulated in LVNC heart tissue, SORBS2 may be associated with this disease. The proteins that are highly correlated with SORBS2 may play a certain role in the occurrence of diseases.

### Proteins that interact with SORBS2

**Figure 2:**
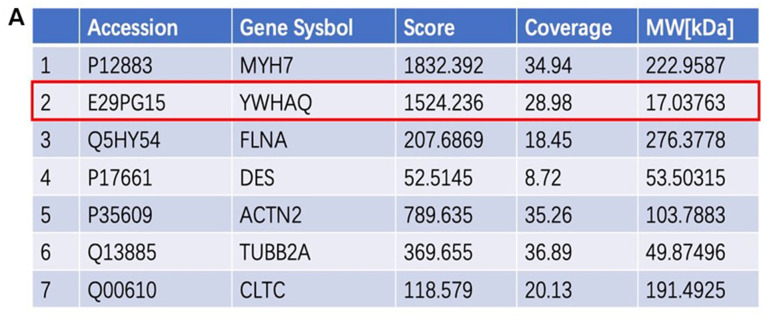
**Proteins that interact with SORBS2.** (**A**) The proteins interacting with SORBS2 were screened according to the evaluation score and reliability analysis. We focused on YWHAQ interacting with SORBS2.

Some proteins interacting with SORBS2 were found by preliminary screening, and co-IP was further used to verify the interaction between them and SORBS2. For several proteins we focused on, the immunofluorescence technique was used for detection in hESC-CMs to re-confirm that the related proteins interacted with SORBS2. The proteins interacting with SORBS2 were screened by bioinformatics and online websites. In addition, 6 proteins highly correlated with SORBS2 were screened by IP: MYH7, YWHAQ, DES, ACTN2, TUBB2A and CLTC (Figure 2A).

According to previous reports, TUBB2A does interact with SORBS2 [9]; moreover, 14-3-3 family knockout mice can have the phenotype of LVNC disease [15]. SORBS2 interacts with YWHAB and YWHAZ. Both YWHAB and YWHAZ belong to the 14-3-3 family of proteins, of which YWHAE (14-3-3) has been reported to be associated with the LVNC phenotype. In E12.5 embryonic mice, 14-3-3 deletion showed incomplete densification. Therefore, we focused on the 14-3-3 family as a protein highly correlated with SORBS2 (Figure 2A). These data suggest that YWHAQ, as a protein that interacts with SORBS2, may be involved in the pathogenesis of related diseases.

### Embryonic stem cells induced to differentiate into cardiomyocytes

**Figure 3:**
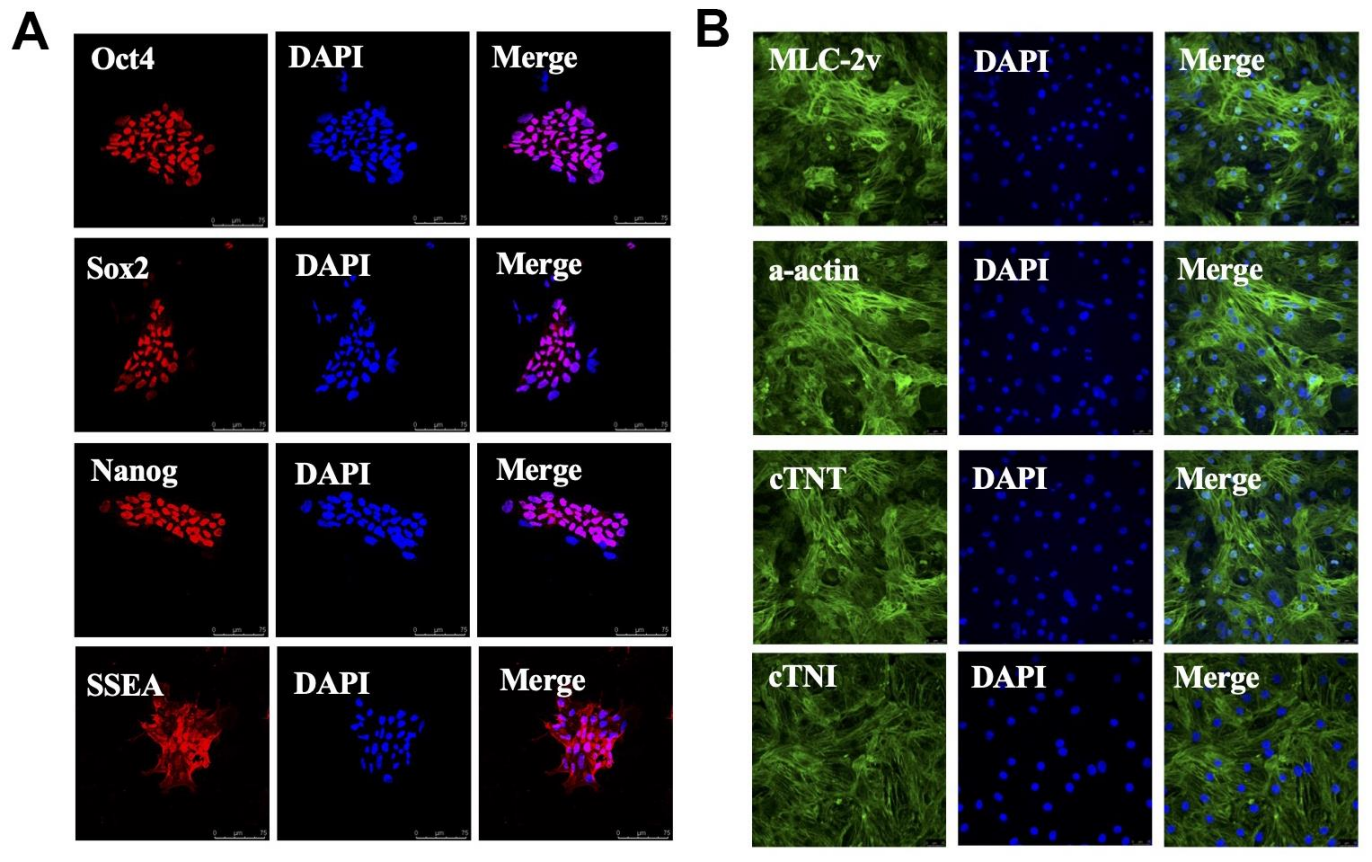
**Stem cells are induced to differentiate into cardiomyocytes.** (**A**) Human embryonic stem cells (hESC) expressing Oct4, Sox2, Nanog and SSEA. (**B**) hESC-derived cardiomyocytes (hESC-CMs) expressing MLC-2v, α-actinin, cardiac troponin-I (cTnI) and cardiac troponin-T (cTnT). Scale bars=25μm.

Next, we used hESC-CMs as the carrier in *in vitro* experiments to study whether overexpressed SORBS2 would affect cell phenotype and function. The totipotency of human embryonic stem cells was demonstrated by immunofluorescence staining with four stem cell marker proteins: Oct4, Sox2, Nanog and SSEA (Figure 3A). This experiment indicated that hESC still retained stem cell totipotency and could be used in the following induction experiments.

The induced cardiomyocytes showed high purity and continuous beating. Four myocardial cell protein markers, MLC-2V, α-actinin, cardiac troponin I (cTnI) and cardiac troponin T (cTnT), were detected by immunofluorescence staining. This indicated that the induction of differentiation into cardiomyocytes was successful and that the purity of the cardiomyocytes was relatively high (Figure 3B).

### SORBS2 interacts with YWHAQ

**Figure 4:**
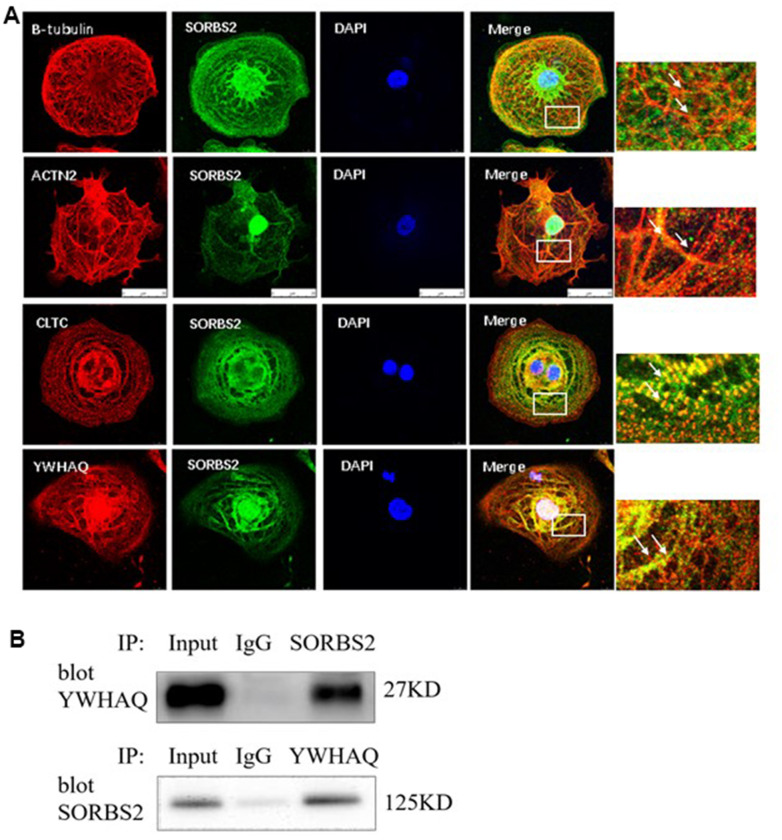
**SORBS2 interacts with YWHAQ.** (**A**) To better understand β-tubulin, ACTN2, CLTL and YWHAQ, we next performed studies in hESC-derived cardiomyocytes (hESC-CMs). Immunofluorescence revealed a clear colocalization of SORBS2 with β-tubulin, ACTN2, CLTL and YWHAQ in the hESC-CMs. The SORBS2 protein (red) colocalized with β-tubulin (green), and the merged images are shown in yellow. (**B**) Extracted proteins from the cardiomyocytes ware untreated (input) or precipitated by an anti-Flag or anti-Myc antibody and then separated by 4-12% SDS-PAGE. Co-immunoprecipitation (Co-IP) of the heart tissue showing SORBS2 binding with YWHAQ.

We further determined the localization of the selected proteins and the expression location of SORBS2 in the induced cardiomyocytes using immunofluorescence staining. We found that β-tubulin, ACTN2, CLTC and YWHAQ showed co-localization with SORBS2 (Figure 4A). Meanwhile, we used the co-immunoprecipitation assay to verify the interaction between SORBS2 and YWHAQ. These data collectively demonstrated that SORBS2 interacted with YWHAQ in normal myocardium (Figure 4B).

**Figure 5:**
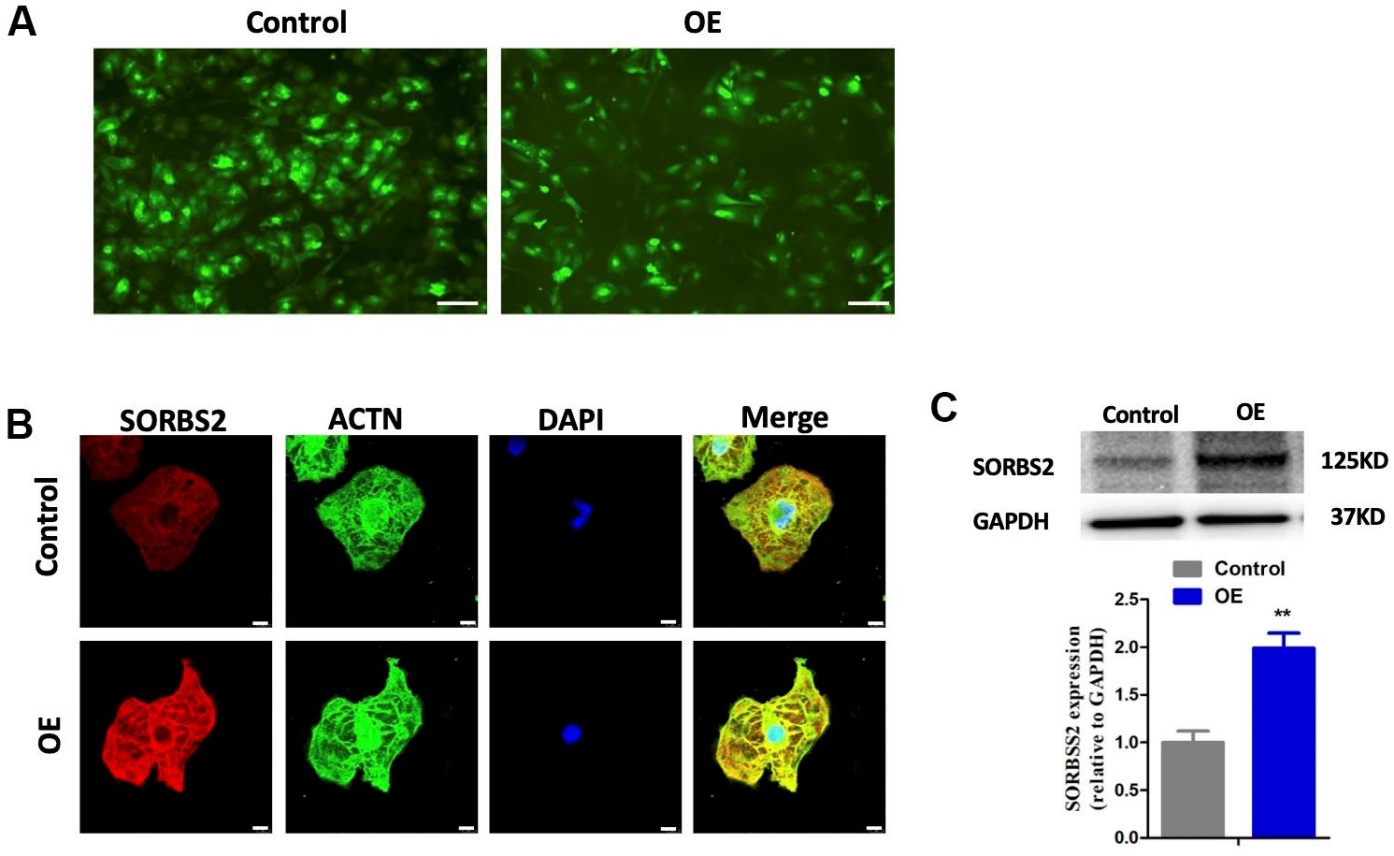
**Expression of SORBS2 in hESC-CMs transfected with HBLV-h-SORBS2 was higher compared with the control groups.** (**A**) Cardiomyocytes transfected with lentivirus. (**B**) Representative images of SORBS2 in hESC-CMs transfected with empty virus (control) and HBLV-h-SORBS2 (SORBS2-OE). (**C**) Western blotting detection of the expression levels of SORBS2 in overexpression group and control group. The protein level of SORBS2 was normalized to GAPDH for each sample. Statistical analysis of the expression of SORBS2 in hESC-CMs transfected with empty virus (control) and HBLV-h-SORBS2 (SORBS2-OE) (n =3 per group, * p < 0.05; Student's t-test). Data are shown as the mean ± SEM.

**Figure 6:**
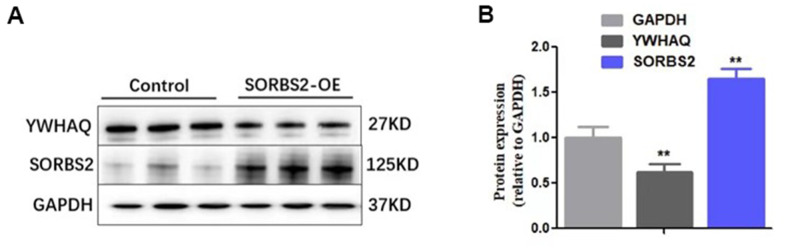
**YWHAQ protein decreased in the group that overexpressed SORBS2.** (**A**) The expression of YWHAQ inf immunoblots of hESC-derived cardiomyocytes (hESC-CMs). (**B**) Statistical analysis of the expression of YWHAQ in hESC-CMs transfected with HBLV-h-SORBS2 and control cells. (* *p < 0.01).

The hESC-CMs were transfected with HBLV-h-SORBS2 lentivirus to induce overexpression of SORBS2 (Figure 5A). Figure 5B shows that the levels of SORBS2 were significantly increased in the hESC-CMs in which SORBS2 showed overexpression by immunofluorescence staining. The immunoblots showed an increased proportion of SORBS2 (Figure 5C). More dramatically, to better understand the relationship between SORBS2 and YWHAQ in hESC-CMs in which SORBS2 was overexpressed, we found decreased YWHAQ expression (Figure 6A, 6B). Our previous studies showed that as a specific up-regulated protein in LVNC heart tissue, SORBS2 may have a particular relationship to this disease. Moreover, as the protein YWHAQ has a strong SORBS2 correlation, it would undoubtedly provide a more favorable research basis in our future studies.

## DISCUSSION

In the present study, the major findings were the following:

1. The protein SORBS2 was specifically overexpressed only in LVNC and may have some relationship with this disease. For the first time, we used normal myocardial tissue to conduct IP experiments on SORBS2 and screened the interacting proteins highly correlated with SORBS2.
2. SORBS2 overexpression caused decreased YWHAQ, and SORBS2 and YWHAQ were co-expressed in hESC-CMs and myocardial tissue. SORBS2 co-immunoprecipitated with YWHAQ in hESC-CMs. The interaction between SORBS2 and YWHAQ protein in both position and function suggests that these proteins together have an important biological effect.

The pathogenesis of LVNC involves various factors such as embryonic development and genetic variation [13, 16]. During the development of the heart, there is cell cycle arrest due to the genetic abnormality, cell development is blocked and the densification process is impaired. The muscle trabecular space causes protuberances to persist, resulting in the cavernous ventricular muscle on the endocardial side.

The sarcoplasmic protein SORBS2 is an adapter protein and a member of the SOHO protein family. It is widely expressed in human tissues, especially highly expressed in cardiomyocytes, and located in the Z band of mature muscle fibers [9, 10]. SORBS2 is considered a scaffold protein that plays an important role in the stability of the cytoskeleton, which maintains cell morphology and regulates cell movement, so as to coordinate the physiological functions of cells. The cytoskeleton coordinates multiple signaling pathways that converge on actin and microtubule cytoskeletons [11]. The main function of SORBS2 is to maintain the structural integrity of myocardial cells and participate in cell adhesion and signal transduction. The overexpression of SORBS2 can lead to defects in cytoskeleton dynamics and is related to the disorder of microtubule skeleton stability [11]. It has been reported that tubulin polymerization or depolymerization can regulate myocardial polarity [12], oriented cell division (OCD) [13] and oriented migration of myocardial cells [14], which are highly correlated with muscle trabecular initiation and formation. Taken together, our findings provide unique insights into the relationship SORBS2 has with YWHAQ, which leads to cell cycle changes in the disease LVNC.

Among the interaction proteins selected, tubulin and ACTN, cytoskeleton proteins, have a biological interaction with SORBS2, which has been reported by others. First, it proves the reliability of IP in our experiment; second, it is an extremely complex process to affect the occurrence of a disease. We want to find proteins biologically related to SORBS2 from other aspects to explore the mechanism of LVNC. To further explore the mechanism of disease occurrence and development. Actinin and tubulin, as structural protein of heart muscle plays an important role in maintaining the skeletal structure of the myocardium. Therefore, as a cardiac cytoskeletal protein, SORBS2 may have some relationship with the common cardiac structural proteins actinin and tubulin. We validated a previous study [17] that presented a new heart disease mechanism, where microtubule densification-mediated redistribution of JP2 within the membrane system may be associated with heart failure. Others have reported that the expression of JP2 is markedly reduced in diseased hearts [18–21]. Our previous data are consistent with the new mechanism; we identified densification of microtubules as a cause of the redistribution of JP2, but this was not decreased in LVNC human cardiac tissue and in hESC-CMs with overexpressed SORBS2 [9].

Previous studies have shown that down-regulation of 14-3-3 protein can promote the development of LVNC [15]. 14-3-3 is widely expressed in embryonic heart tissue. Studies have shown that 14-3-3 protein deficiency can affect the expression of cyclin E1 and p27Kip1, which are important genes in cell cycle regulation, leading to the arrest of cardiomyocyte development and the eventual occurrence of cardiac insufficiency [15]. The previous study found that SORBS2 could interact with 14-3-3 proteins. In addition, 14-3-3 protein was significantly down-regulated in the myocardial tissues with high SORBS2 expression. Therefore, we speculated that the high expression of SORBS2 could affect the development of cardiomyocytes through the down-regulation of 14-3-3 protein, thus leading to the occurrence of LVNC.

In normal embryonic heart development, at 0~4 weeks, coronary circulation has not formed; rather it is ventricular through a large number of trabecular muscles interwoven into a network structure, trabecular muscle gap and left ventricular cavity, to maintain the blood supply of the embryonic heart, similar to blood supply in cold-blooded animals, where the heart muscle at this time is a cavernous structure. At 5-6 weeks of embryonic development, subendocardium densification occurs, and the trabecular space (crypt) densifies into capillaries, forming a coronary microcirculation system, and finally only a small amount of subendocardium trabecular muscle remains [11]. Since the densification of the myocardium in the right ventricle is earlier than that in the left ventricle, densification in the lower heart is earlier than that in the apex, and the epicardium is earlier than the endocardium, where the densification of myocardium mostly occurs in the left ventricle, the intimal side from the middle of the ventricle to the apex of the heart. On the basis of our previous results and existing research reports, we propose the following hypothesis that SORBS2 can regulate the cell cycle by activating 14-3-3 protein expression, thereby promoting the development of LVNC. We will combine the previously published results and previous research basis and use the overexpressed SORBS2 cell model as the research focus to explore the role of SORBS2 in the pathophysiological process of LVNC, so as to explore diagnostic strategies and therapeutic targets in LVNC.

Several limitations should be considered in the present study. First, because our experiments were confined to hESC-CMs (*in vitro*) and subjected to virus-mediated SORBS2 overexpression, future studies should focus on mice (*in vivo*) with enhanced overexpression of SORBS2 to determine whether they exhibit typical phenotypes of LVNC. Second, although SORBS2 interacts with YWHAQ and lowers its expression, the detailed mechanism still needs to be studied.

In summary, our study, based on mass spectrometry identification of proteins in human heart tissues, revealed that SORBS2 is especially associated with YWHAQ. We confirmed that overexpression of SORBS2 in hESC-CMs clearly decreased YWHAQ and caused cell cycle impairment, eventually leading to cardiomyocyte dysfunction. Our findings thus revealed that SORBS2 interacts with YWHAQ, which may lead to LVNC disease.
